# Distribution of the causes of fever of unknown origin in China, 2013-2022

**DOI:** 10.2478/jtim-2024-0008

**Published:** 2024-07-27

**Authors:** Sihan Kang, Rui Zheng

**Affiliations:** Department of Pulmonary and Critical Care Medicine, Shengjing Hospital of China Medical University, Shenyang 110004, Liaoning Province, China

**Keywords:** fever of unknown origin, infections, autoimmune diseases, neoplastic diseases, adult-onset Still's disease, human immunodeficiency virus infections, tuberculosis

## Abstract

**Background and Objectives:**

Fever of unknown origin (FUO) has long been a cause for concern among clinicians, and its spectrum has evolved with progress in medicine. This study aimed to investigate the distribution of causes of FUO in China between 2013 and 2022 to facilitate the clinical understanding of the etiology of FUO.

**Materials and Methods:**

Case series of FUO in China published between 2013 and 2022 were retrieved from PubMed, Wanfang Data, and CNKI databases and retrospectively analyzed. The rates of different causes of FUO were calculated, and these data were compared with previously published distributions of causes of FUO in China.

**Results:**

The causes of FUO with the highest rates from the 51 identified case series (*n* = 19,874) were infectious, autoimmune, and neoplastic diseases (59.6%, 14.3%, and 7.9%, respectively). A comparison of a subset (43 case series subdivided by disease category, *n* = 16,278) with previously reported data revealed an increased rate of FUO attributed to infectious diseases in the past decade, with a significantly higher rate attributed to bloodstream infections (10.0% *vs*. 4.8%) and a significantly lower rate attributed to tuberculosis (9.3% *vs*. 28.4%), compared with the rates from the previous period. In contrast, the rates of FUO attributed to both autoimmune and neoplastic diseases decreased, with significantly decreased rates attributed to adult-onset Still's disease among autoimmune diseases (4.6% *vs*. 8.5%) and lung cancer among neoplastic diseases (0.6% *vs*. 1.6%).

**Conclusion:**

Despite an overall increase in the rate attributed to infectious diseases, that attributed to tuberculosis has decreased. The rates attributed to both autoimmune and neoplastic diseases have also decreased.

## Introduction

As a common symptom in clinical practice, fever often presents as the first disease manifestation and may even be the only symptom. Among such presentations, fever of unknown origin (FUO) has long been a cause for concern among clinicians. The diagnostic criteria for FUO were established by Petersdorf and Beeson as “fever (temperature > 38.3°C on several occasions) for over 3 weeks with no cause identified after hospitalization and examination for 1 week”.^[1]^ In 1991, Durack and Street revised these criteria to “fever (temperature > 38.3°C on several occasions) for over 3 weeks with no cause identified after hospitalization for 3 days or after 3 outpatient visits” and classified FUO into four categories-classical, nosocomial, neutropenic, and human immunodeficiency virus/acquired immunodeficiency syndrome (HIV/AIDS)-associated FUO.^[2]^ In certain patients, FUO has a relatively narrow spectrum of underlying causes due to specific underlying health conditions, and a clinical diagnosis can be made more easily. However, the diagnosis of classical FUO is more difficult, as the patient may have been healthy before fever onset, only have a subclinical disease, or have a chronic disease.

The causes of FUO have changed substantially over the past century.^[3]^ Objective factors including geographical and environmental differences and a country’s developmental stage can result in very different causes of FUO.^[4]^ The range of causes of FUO is constantly changing due to factors including advances in diagnostic techniques, the use of novel drugs (*e.g*., immunosuppressants), and climate change.^[4]^ Therefore, we conducted a systematic review of case series reported during the period 2013-2022 to analyze the distribution of causes of FUO in China and to compare it with previously reported distributions to understand the differences, which may facilitate clinical decision-making.

## Materials and methods

### Literature search strategy

PubMed was searched for articles published in the English language between January 2013 and December 2022 using ‘(fever of unknown origin) or (FUO) or (pyrexia of unknown origin) or (PUO)’ and (China) as the MeSH and search terms. CNKI and Wanfang database was searched for articles published Chinese language within the same date range using (fever of unknown origin) or (fever pending investigation) as the search terms.

### Data selection

Inclusion criteria: case series that met one of the two diagnostic criteria for FUO,^[1,2]^ included the distribution of causes of FUO, and involved Chinese individuals.

Exclusion criteria: case series that enrolled only children or collected data from a specialty, not a general hospital.

### Statistics

Data were manually extracted by researchers for further analysis. The distributions of causes of FUO between current and previous report periods were compared using the chi-square test using SPSS 27.0 software (IBM, Armonk, NY, USA). Statistical significance was set at *P* < 0.05.

## Results

### Data retrieval

In the initial database search, 2604 publications were identified. After screening for publication type and removing duplicates, 583 articles with FUO cases remained. After excluding 486 articles based on titles or abstracts, 97 case series were selected for further analyses. Among these, 46 were excluded because they were conducted at specialty hospitals or focused solely on children. Finally, 51 articles with 19,874 patients were analyzed, of which 43 articles characterized their 16,278 patients by disease subcategories.

### Distribution of causes of FUO in 2013-2022

In the 51 included case series (*n* = 19,874), causes of FUO were infectious diseases in 11,837 cases (59.6%), autoimmune diseases in 2850 cases (14.3%), neoplastic diseases in 1562 cases (7.9%), other diseases in 1197 cases (6.0%), and undiagnosed cause in 2428 cases (12.2%). Thus, infectious diseases accounted for the majority of FUO cases. In the 43 case series subdivided by disease (*n* = 16,278), infectious diseases were again the primary cause in 9766 cases (60.0%). The top three infectious diseases were respiratory infections in 2192 cases (13.5%), bloodstream infections in 1632 cases (10.0%), and tuberculosis in 1509 cases (9.3%). Among the 2328 FUO cases attributed to autoimmune diseases (14.3%), the top three causes were adult-onset Still’s disease (AOSD) in 753 cases (4.6%), systemic lupus erythematosus in 351 cases (2.2%), and vasculitis in 290 cases (1.8%). Neoplastic diseases were responsible for 1331 FUO cases (8.2%); the top three causes were lymphoma in 535 cases (3.3%), leukemia in 207 cases (1.3%), and gastrointestinal tumors in 182 cases (1.1%). Among other diseases in 951 FUO cases (5.8%), the top three causes were lymphadenitis in 224 cases (1.4%), subacute thyroiditis in 213 cases (1.3%), and drug fever in 144 cases (0.9%).

### Distributions of causes of FUO in 2013-2022 compared with those in earlier years

**Figure 1:**
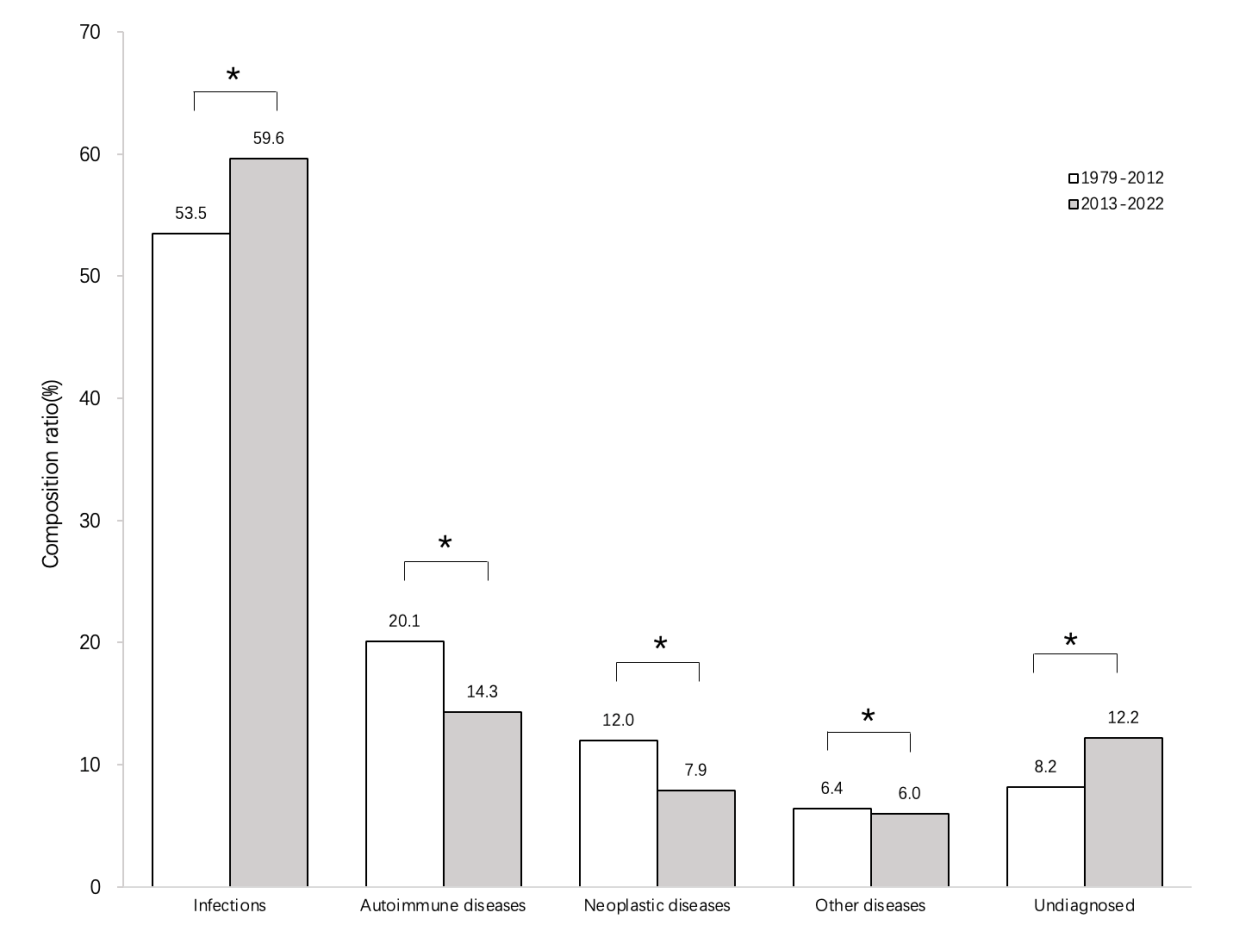
Graph showing the distribution of FUO disease constituent categories from 1979 to 2012 and from 2013 to 2022. *P < 0.05. FUO: fever of unknown origin.

Comparison of major disease spectrum classifications The distributions of causes of FUO were compared between the case series in the present study (51 case series, *n* = 19,874) and those in the article “Chinese literature review of etiology distribution of adult patients with fever of unknown origin from 1979 to 2012” (43 case series, *n* = 10,201, Table 1).^[5]^ The rate attributed to infectious diseases significantly increased in the present case series compared with those from 1979 to 2012 (59.6% *vs*. 53.5%, respectively), whereas the rates attributed to autoimmune, neoplastic, and other diseases significantly decreased (14.3% *vs*. 20.1%, 7.9% *vs*. 12%, and 6.0% *vs*. 6.4% respectively), and the rate attributed to undiagnosed causes significantly increased (12.2% *vs*. 8.2%, Figure 1).

**Table 1:** Distribution of the different disease categoriesin 1979-2012 and 2013-2022

Disease categories	1979-2012	(% of total)	2013-2022	(% of total)	*P*-value
Infections	5458	53.5	11837	59.6	< 0.001
Autoimmune causes	2050	20.1	2850	14.3	< 0.001
Neoplastic diseases	1204	12.0	1562	7.9	< 0.001
Other diseases	653	6.4	1197	6.0	< 0.001
Undiagnosed	836	8.2	2428	12.2	< 0.001

Case series 1979-2012 had 10,201 cases in 43 papers and case series 2013-2022 had 19,874 cases in 51 papers; Statistical significance is examined by using χ2-test to assess the difference of constituent ratios in the two periods; *P* < 0.05 is considered statistically significant.

#### Comparison of causes of FUO subdivided by disease after 2000

With the rapid development of medical care in China since 2000, the quality of medical testing and diagnostic procedures has substantially improved, and the range of causes of FUO in case series reported after 2000 is relatively comprehensive. Therefore, the data reported by Tan et al.^[5]^ were recompiled, including only the period 2000-2012 (*n* = 7747). These data were compared with those from the 43 case series subdivided by disease (*n* = 16,278). The results showed that the rate of FUO attributed to infectious diseases has increased in the last decade, among which the rates attributed to bloodstream infections (10.0% *vs*. 4.8%), urinary tract infections (4.4% *vs*. 2.1%), and HIV/AIDS (0.9% *vs*. 0.6%) significantly increased, compared with the rates reported in earlier years; however, the rate attributed to tuberculosis significantly decreased (9.3% *vs*. 28.4%). Among autoimmune diseases, the rates of causes in all subcategories significantly decreased, compared with those reported in earlier years, including those attributed to AOSD (4.6% *vs*. 8.5%), systemic lupus erythematosus (2.2% *vs*. 2.8%), rheumatoid arthritis (0.7% *vs*. 1.0%), vasculitis (1.8% *vs*. 2.0%), and undifferentiated connective tissue disease (1.0% *vs*. 1.2%). Among neoplastic diseases, there were significant decreases in the rates attributed to lung cancer (0.6% *vs*. 1.6%) and lymphoma (3.3% *vs*. 3.9%) and a significant increase in that attributed to leukemia (1.3% *vs*. 0.9%). Among other diseases, the rate attributed to drug fever (0.9% *vs*. 1.8%) significantly decreased, whereas those attributed to (necrotizing) lymphadenitis (1.4% *vs*. 1.1%) and subacute thyroiditis (1.3% *vs*. 0.3%) significantly increased (Table 2).

**Table 2:** Distribution of the different disease categories in 2000-2012 and 2013-2022

Disease categories	2000-2012	(% of total)	2013-2022	(% of total)	*P*-value
**Infections**	3959	51.1	9766	60.0	<0.001
Blood stream infection	372	4.8	1632	10.0	<0.001
Tuberculosis	2200	28.4	1509	9.3	<0.001
Urinary tract infection	163	2.1	717	4.4	<0.001
Infective endocarditis	116	1.5	258	1.6	0.608
HIV/AIDS	46	0.6	153	0.9	0.006
Fungal infection	108	1.4	134	0.8	<0.001
Others	954	12.3	5363	32.9	<0.001
**Autoimmune causes**	1704	22.0	2328	14.3	<0.001
AOSD	658	8.5	753	4.6	<0.001
SLE	217	2.8	351	2.2	0.002
Rheumatoid arthritis	77	1.0	108	0.7	0.006
Vasculitis	155	2.0	290	1.8	0.239
UCTD	93	1.2	160	1.0	0.123
Others	504	6.5	666	4.1	<0.001
**Neoplastic diseases**	852	11.0	1331	8.2	<0.001
Lymphoma	302	3.9	535	3.3	0.016
Leukemia	70	0.9	207	1.3	0.012
Lung cancer	124	1.6	90	0.6	<0.001
Others	356	4.6	499	3.1	<0.001
**Other Diseases**	542	7.0	951	5.8	<0.001
(Necrotizing) lymphadenitis	85	1.1	224	1.4	0.073
Subacute thyroiditis	23	0.3	213	1.3	<0.001
Drug fever	139	1.8	144	0.9	<0.001
Others	295	3.8	370	2.3	<0.001
**Undiagnosed**	689	8.9	1902	11.7	<0.001

Case series 2000-2012 had 7747 cases and that of 2013-2022 had 16,278 cases in 43 case series; Statistical significance is examined by using χ^2^-test to assess the difference of constituent ratios in the two regions; P < 0.05 is considered statistically significant. HIV/AIDs: human immunodeficiency virus/acquired immunodeficiency syndrome; AOSD: adult-onset Still’s disease; SLE: systemic lupus erythematosus; UCTD: undifferentiated connective tissue disease.

### Geographical differences in the distribution of causes of FUO in 2013-2022

Regarding the city of publication, using the Qinling-Huaihe Line as the dividing line between North and South China, 24 case series (*n* = 10,114) were conducted in North China and 27 case series (*n* = 6164) in South China. The rate of FUO attributed to infectious diseases was significantly higher in North China (65.5% *vs*. 51.0%), whereas those attributed to autoimmune (17.2% *vs*. 12.5%) and neoplastic (11.0% *vs*. 6.4%) diseases were significantly higher in South China (Table 3).

**Table 3:** Composition of cases of unexplained fever etiology by geographic region

Disease categories	Northern cases	(% of total)	Southern cases	(% of total)	*P*-value
**Infectious diseases**	6625	65.5	3141	51.0	<0.001
Bloodstream infection	1125	11.1	498	8.1	<0.001
Urinary tract infection	559	5.5	158	2.6	<0.001
Infective endocarditis	146	1.4	112	1.8	0.064
Respiratory infection	1722	17.0	470	7.6	<0.001
Digestive system infection	296	2.9	147	2.4	0.039
CNS Infection	90	0.9	60	1.0	0.588
SSTIs	6	0.1	13	0.2	0.006
Tuberculosis	763	7.5	746	12.1	<0.001
Brucellosis	479	4.7	32	0.5	<0.001
EBV	163	1.6	163	2.6	<0.001
CMV	67	0.7	59	1.0	0.037
RSV	13	0.1	4	0.1	0.223
HIV/AIDS	79	0.8	74	1.2	0.007
Hepatotropic virus	15	0.1	15	0.2	0.17
Mycoplasma infection	67	0.7	35	0.6	0.458
Fungal infection	68	0.7	66	1.1	0.006
Rickettsioses	29	0.3	3	0.0	<0.001
Parasite	17	0.2	26	0.4	0.002
Typhoid fever	41	0.4	34	0.6	0.182
Others	880	8.7	426	6.9	<0.001
**Autoimmune causes**	1265	12.5	1063	17.2	<0.001
AOSD	374	3.7	379	6.1	<0.001
SLE	193	1.9	158	2.6	0.005
Rheumatoid arthritis	59	0.6	48	0.8	0.135
Sjögren’s syndrome	53	0.5	50	0.8	0.025
HPS	57	0.6	22	0.4	0.066
Vasculitis	170	1.7	120	1.9	0.213
PMR	30	0.3	25	0.4	0.245
Polymyositis	30	0.3	26	0.4	0.186
Behcet syndrome	19	0.2	7	0.1	0.25
Ankylosing spondylitis	30	0.3	13	0.2	0.301
Polymyositis	20	0.2	15	0.2	0.542
Reactive arthritis	43	0.4	15	0.2	0.059
UCTD	69	0.7	91	1.5	<0.001
Others	118	1.2	94	1.5	0.051
**Neoplastic diseases**	652	6.4	679	11.0	<0.001
Leukemia	85	0.8	122	2.0	<0.001
Lymphoma	224	2.2	311	5.0	<0.001
Multiple myeloma	25	0.2	39	0.6	<0.001
MDS	14	0.1	7	0.1	0.668
Gastrointestinal cancer	108	1.1	74	1.2	0.435
Lung cancer	55	0.5	35	0.6	0.841
Others	141	1.4	91	1.5	0.668
**Other diseases**	506	5.0	445	7.2	<0.001
(Necrotizing) lymphadenitis	141	1.4	83	1.3	0.8
Subacute thyroiditis	146	1.4	67	1.1	0.052
Hypereosinophilia	5	0.0	4	0.1	0.684
Drug fever	52	0.5	92	1.5	<0.001
Functional fever	11	0.1	13	0.2	0.099
Others	151	1.5	186	3.0	<0.001
**Undiagnosed**	1066	10.5	836	13.6	<0.001

There were 16,278 cases in 43 papers, including 10,114 cases in the North and 6164 cases in the South; Statistical significance is examined by using χ^2^-test to assess the difference of constituent ratios in the two regions; *P* < 0.05 is considered statistically significant. CNS: central nervous system; SSTIs: skin and soft tissue infections; EBV: Epstein-Barr virus; CMV: cytomegalovirus; RSV: respiratory syncytial virus; HIV/AIDs: human immunodeficiency virus/acquired immunodeficiency syndrome; AODS: adult onset Still’s disease; SLE: systemic lupus erythematosus; HPS: hemophagocytic syndrome; PMR: polymyalgia rheumatica; UCTD: undifferentiated connective tissue disease; MDS: Myelodysplastic syndromes.

## Discussion

The distribution of causes of FUO in China between 2013 and 2022 was analyzed and compared with previously reported data. The overall pattern did not change substantially, with infectious diseases still accounting for most FUO cases, followed by autoimmune diseases, and other diseases accounting for the lowest rates. However, comparing causes of FUO by disease showed some changes over time.

The rate of FUO attributed to infectious diseases has increased in the last decade possibly due to the increased incidence of cancer and the consequently increased rate of immunosuppression, leading to increased rates of infections. This may also be associated with recent improvements in diagnostic procedures: fevers caused by autoimmune and neoplastic diseases are diagnosed at earlier stages and are no longer classified as FUO. Although the overall proportion of FUO cases attributed to infectious diseases increased, different infectious agents exhibited different trends.

Compared with the rates during the period 2000-2012, the rates attributed to tuberculosis have significantly decreased in the last decade, possibly due to two major reasons. First, China has implemented measures that have decreased the annual rate of new tuberculosis infections.^[6]^ Second, diagnostic techniques have substantially improved, including interferon-gamma release assays, GeneXpert MTB/RIF, and high-throughput second-generation sequencing, which have facilitated tuberculosis diagnosis at earlier stages.^[7,8]^ Even with this decrease, tuberculosis remains a leading cause of FUO primarily because atypical mycobacterial disease and extrapulmonary tuberculosis differ from typical tuberculosis in clinical presentation, delaying the diagnosis.^[9]^ Moreover, the use of antibiotics that are effective against both common bacterial infections and tuberculosis (*e.g*., fluoroquinolones) may lead to delayed diagnosis of lung infections with an unidentifiable causative pathogen, requiring differentiation through treatment. In such cases, antibiotics that are effective against bacteria but not tuberculosis (*e.g*., β-lactam antibiotics) should be used. In 2021, the world health organization (WHO) declared China the country with the second largest tuberculosis burden worldwide. Thus, clinicians should consider tuberculosis when encountering patients with FUO.

Our study revealed that the prevalence of HIV/AIDS among FUO cases increased in the past decade, which may also be related to recent improvements in testing techniques. Both the prevalence and mortality rates of HIV/AIDS in China have continuously increased in recent years, consistent with our finding that the rate of FUO attributed to HIV/AIDS has increased. The rates of heterosexual and homosexual HIV/ AIDS transmission have respectively increased from 48.3% and 9.1% to 74.2% and 23.3% between 2009 and 2020.^[10]^ At the early stages of HIV infection, the acute retroviral syndrome may cause fever; however, HIV infection cannot be diagnosed using antibody tests, so the fever may be categorized as FUO. Thus, clinicians should be more vigilant when treating patients in high-risk groups and pay attention to accompanying symptoms. Early diagnosis can be achieved through detailed ascertainment of medical history and clinical examination. In patients with late-stage HIV infection, CD4^+^ T cells are severely depleted, and opportunistic infections by Mycobacterium tuberculosis, cytomegalovirus, fungi, and other pathogens are the primary causes of prolonged fever. The pathogen type may be correlated with CD4^+^ T cell counts.^[11]^ Growing sex differences in HIV/AIDS deaths in China have been reported. Between 1990 and 2008, the HIV/ AIDS mortality rate was twice as high in men as in women. After 2008, this gap widened, and by 2016, the mortality rate in men was almost three times that in women.^[10]^ According to the National Center for AIDS/ STD Control and Prevention, and Chinese Center for Disease Control and Prevention, the proportion of newly reported HIV infection cases among men aged ≥ 60 years increased from 7.41% in 2010 to 18.21% in 2020, with most infections occurring through heterosexual contacts. Therefore, clinicians should be aware of HIV transmission among men and older adults, who may not have been screened at primary hospitals; it should not be neglected in patients with FUO.

We also found that the proportion of FUO cases attributed to bloodstream infections has increased recently, possibly because an increasing number of invasive procedures (*e.g*., indwelling central venous catheters, urinary catheters, thoracic/abdominal catheters, and dialysis treatments) have become more widespread.^[12,13]^ A study from Spain revealed an increased frequency of bloodstream infections. ^[14]^ Other studies suggest that this increased incidence may be associated with an increase in the number of blood cultures performed, the continuous improvement of blood culture techniques, or the prolonged use or improper care of catheters.^[15, 16, 17]^ These invasive procedures have made treatment more convenient, but mitigating the associated infections and other complications remains a concern for future development. In FUO patients with invasive catheter placement, diagnosis requires a focus on fever due to bloodstream infection.

Fever is a common symptom of both hematologic malignancies and solid tumors. The pathophysiological mechanism may be associated with the release of pyrogenic cytokines.^[18]^ The proportion of FUO cases attributed to neoplastic diseases has significantly decreased now compared with earlier years, which is undoubtedly associated with improvements in testing methods leading to earlier diagnosis of neoplastic diseases (particularly solid cancers). Recently, the use of ^18^fludeoxyglucose-18 positron emission tomography (FDG-PET/CT) has been particularly valuable in the diagnosis of tumors, especially in the identification of potential tumors and tumors in problematic locations.^[19, 20, 21, 22]^ This is consistent with the decreased rate of FUO attributed to lung cancer confirmed in the present study. The naproxen test may be useful in differentiating neoplastic from non-neoplastic fever. This was first described in 1984 when 14 of 15 patients with neoplastic fever exhibited rapid and sustained remission after naproxen treatment, whereas five patients with infectious fever did not, demonstrating that naproxen treatment can prevent fever in patients with neoplasms.^[23]^ A recent meta-analysis showed a 94.1% success rate of neoplastic fever suppression with naproxen.^[24]^ Therefore, naproxen can be used for adjunctive diagnosis in patients with suspected neoplastic fever in which the tumor site is difficult to access. Among FUO-causing diseases, the rate of cases attributed to lymphoma has significantly decreased now compared with earlier years, consistent with international reports.^[25]^ This may be due to medical advances like ^18^FDG-PET/CT, extranodal tissue biopsy, and lymph node biopsy, which have increased the lymphoma diagnosis rate.^[26]^ However, lymphoma remains a major cause of FUO.^[25]^ The pathogenesis of lymphoma is complex, and early diagnosis before lymphoma development (*e.g*., Epstein-Barr virus infection) is difficult. Low positive rates of lymph node biopsies and limited accuracy of pathologic diagnosis also affect the diagnosis of lymphoma during its development. Therefore, high rates of FUO remain attributed to lymphoma, warranting continued clinical attention.

Drug fever is usually a diagnosis of exclusion, with a broad differential diagnosis that includes all other causes of fever. Drug fever lacks a specific fever pattern, making a definitive clinical diagnosis difficult.^[27,28]^ For patients with suspected drug fever, a detailed medical history and a careful analysis of clinical presentation and laboratory findings are essential, as these can help exclude other differential diagnoses. The present study revealed a decrease in the rate of FUO attributed to drug fever, which may be related to increased awareness of drug fever among clinicians and the fact that other diseases can be ruled out at early stages due to improvements in medical technology. Antibiotics are the most common drugs in the treatment of infectious fevers but are also the most common cause of drug fever, accounting for about one-third of all cases, with β-lactams and sulfonamides being the most commonly associated antibiotics.^[29]^ Drug fever is difficult to diagnose in patients undergoing treatment with antibiotics for infections. Patients with drug fever are often well enough to not even be aware of the fever despite an elevated body temperature that may be accompanied by progressive leukopenia. In such cases, discontinuing or switching antibiotics may result in defervescence.^[30]^ When antibiotic treatment effectively causes the fever to subside, but the fever reappears and is accompanied by a rash, the clinician should consider drug fever. Timely discontinuation of the causative antibiotic and early diagnosis of drug fever is essential for preventing delayed treatment and prolonged hospitalization. The difficulty in diagnosing drug fever is underscored by a study finding that 15% of cases ultimately diagnosed as drug fever met the definition of FUO.^[31]^ Drug fever is difficult to diagnose not only because it requires the exclusion of other differential diagnoses but also because it is diagnosed retrospectively after drug discontinuation.

The overall rate of FUO attributed to autoimmune diseases has decreased, which may be related to the continuous improvements in diagnostic techniques and criteria enabling earlier diagnoses.^[32]^ AOSD is a very rare disease, and current research has focused on its diagnosis and differential diagnosis. Changes in AOSD incidence have not been reported,^[33]^ and AOSD remains a major cause of FUO. Due to the lack of specific symptoms and ancillary tests, AOSD remains a diagnosis of exclusion, greatly increasing the diagnostic difficulty.^[34]^ Fever is a common symptom of AOSD, and the rate of FUO attributed to AOSD has decreased in recent years. This is consistent with advances in medical testing, which have allowed many cases that would previously have been categorized as FUO to be definitively diagnosed as diseases other than AOSD. Several reports have shown that patients initially diagnosed with AOSD were diagnosed with other diseases (*e.g*., leprosy, Mycoplasma pneumonia) through skin biopsies or serological and other tests.^[35,36]^ Early disease recognition and initiation of treatment are essential in AOSD, which may cause multiple organ dysfunction. A French systematic review suggested that the average time from fever to treatment in patients with AOSD is 3 weeks, and treatment delays could be fatal.^[37]^ Therefore, patients with suspected AOSD should be actively screened for other diseases for early diagnosis and treatment to improve outcomes.

The present study also compared the distributions of causes of FUO between North and South China. The rate of FUO attributed to infectious diseases, especially respiratory infections, was higher in North China, whereas the rate of FUO attributed to autoimmune diseases was higher in South China, possibly due to climatic differences. Winter in North China is dry and cold, whereas South China is more humid and lacks centralized municipal heating facilities in winter.^[38]^ The rate of FUO attributed to brucellosis was significantly higher in North China than in South China. This corresponds to the spatial clustering of brucellosis in China and may be related to the animal husbandry industry primarily located in the north. Therefore, regional differences should be considered when assessing FUO.

The present study has some limitations. Although case series enrolling only children were excluded, case series that included children were considered in this study, which may have affected the rate of FUO in the overall population. Stratified analyses of age groups were impossible because the case series only had specified age ranges.

In conclusion, the top three causes of FUO in China in the past 10 years were infectious, autoimmune, and neoplastic diseases. Overall, the rate attributed to infectious diseases has increased, but that attributed to tuberculosis has significantly decreased. The rates attributed to both autoimmune and neoplastic diseases have decreased, but AOSD and lymphoma warrant continued attention. The range of diseases associated with FUO varies geographically, and individualized analyses that incorporate major local causes can help clinicians improve the diagnostic rates of causes of FUO.
